# Basic life support knowledge in a war-torn country: a survey of nurses in Yemen

**DOI:** 10.1186/s12912-022-00923-0

**Published:** 2022-06-06

**Authors:** Sameer A. Alkubati, Christopher McClean, Rebecca Yu, Bander Albagawi, Salman H. Alsaqri, Mohammed Alsabri

**Affiliations:** 1 Department of Medical Surgical Nursing, College of Nursing, University of Hail, Hail City, Saudi Arabia; 2 Department of Nursing, Faculty of Medicine and Health Sciences, Hodeida University, Hodeida, Yemen; 3 Saba University School of Medicine, The Bottom, Caribbean Netherlands; 4 Al-Thawra Modern General Teaching Hospital, Sana’a City, Yemen; 5 Pediatrics, Brookdale University Hospital Medical Center, Brooklyn, NY 11212 USA

**Keywords:** Knowledge, Nurse, Cardiopulmonary resuscitation

## Abstract

**Background:**

Successful implementation of Basic life support (BLS) is critical to improving survival rates and outcomes, especially among healthcare workers. To our knowledge, there is no available literature pertaining to the level of BLS knowledge of health care professionals in Yemen.

**Methods:**

Data was collected for this cross-sectional descriptive study from June to August 2020, using a 10-item questionnaire related to cardiopulmonary resuscitation (CPR) and BLS, along with questions on socio-demographic characteristics. Participants were nurses in public and private hospitals located in Al-Rahida and Al-dimna cities, Taiz governance and Hodeidah city, Hodeidah governance in Yemen.

**Results:**

Out of 220 distributed questionnaires, 200 were returned with a response rate of 90.9%. More than a half (53.65%) of answer choices for BLS knowledge were correct. There was a significant difference in knowledge score based on level of education where those who had Bachelor degree had more knowledge (*P* = 0.000). Those who said they had received training in CPR or received information about CPR had significantly higher scores than those who did not receive (*P* = 0.000).

**Conclusions:**

BLS knowledge among nurses in Yemen is below an acceptable level to ensure maximum survival in the event of cardiac arrest. Disseminating BLS information and training in a cost effective and efficient manner will provide a large benefit in terms of lives saved with minimal costs.

## Background

Basic life support (BLS) entails the recognition of conditions such as heart attack, sudden cardiac arrest, foreign-body airway obstruction, or stroke and the subsequent administration of cardiopulmonary resuscitation (CPR) and defibrillation with an automated external defibrillator^1^ [1]. The American Heart Association notes that the administration of CPR and defibrillation within the first three to five minutes of collapse can yield survival rates ranging from 49% to as high as 75% [2]. In fact, CPR has been demonstrated to double or triple survival from witnessed sudden cardiac arrest [3–5]. Thus, successful implementation of BLS is critical in improving survival rates and outcomes. By extension, health care workers’ knowledge and awareness about BLS is also essential. However, the attitude and level of knowledge of health care professionals varies worldwide [6, 7]. While the demand for BLS courses continues to rise in developed countries, BLS training in underdeveloped and developing countries is not practiced routinely. For instance, a recent survey in Upper Egypt demonstrated suboptimal and inadequate CPR knowledge among medical students and junior doctors; however, positive attitudes and an eagerness towards training was noted in the participants [8]. Similar results were reported by Saquib et al. in a cross-sectional study of health interns in Saudi Arabia [6]. To our knowledge, there is no available literature pertaining to the level of BLS knowledge of health care professionals in Yemen. Therefore, the aim of the present study is to evaluate BLS knowledge among nursing staff in Yemen.

## Methods

### Design and setting

A descriptive cross-sectional survey was conducted over a 3-month period from June to August 2020, to evaluate the nurses’ knowledge about CPR and BLS at the private and public hospitals in AL-rahida and Al-dimna cties, Taiz governance and Hodeidah city, Hodeidah governance, Yemen. All nurses in emergency and critical departments, both males and females, 18 years of age or more, and graduated more than 6 months who were working in the public and private hospitals in the intended cities were eligible for this study.

### Tool of the study

The questionnaire consisted of two sections. The first section focused on the nurses’ demographic and occupational data such as gender, age, level of education, years of work experience, being trained or educated for CPR. The second part contained 10 questions related to CPR and BLS that were developed by the researchers according to the European Resuscitation Council and the American Heart Association guidelines, 2015 [9, 10].

Each question included four possible answers; one of which was correct and three incorrect answers, one of them is the phrase “I do not know” to avoid guessing from the participants. One score was allocated to the correct answer and 0 for an incorrect one; therefore, the total scores for the questionnaire range from 0–10. Then, the total score of nurses’ knowledge was converted to a percentage, over a range of 0–100%. The questionnaire was translated into Arabic language and then translated back into English to determine the accuracy and precision of the translation. Five experts in the medical and nursing emergency participated in the testing of the questionnaire for content validity. Then, a pilot study was conducted to ascertain the reliability of the questionnaire by distributing it to 20 nurses, which was Cronbach’s alpha (*r* = 0.828).

### Ethical considerations

The study was conducted in an ethical and confidential manner. Participation in the study was voluntary, and informed consent was obtained from participants after explanation of the study objective. The researchers obtained Institutional Review Board (IRB) approval prior to carrying out the survey. The questionnaires were conducted anonymously and collected exclusively for research purposes. There were no personal identifiers on the questionnaires. Participation was voluntary with the right to withdraw at any time. all methods were carried out in accordance with the relevant guidelines and regulations. The study was approved by the institutional review board (IRB) of the Al-Shifa College for Technical and Medical Science (Ref. No. 062020**).**

### Data analysis

The Statistical Packages for Social Sciences (SPSS), version 21.0 (IBM Corp., Armonk, NY, USA) was used for processing and analysis of the collected data. Descriptive statistics such as frequencies, percentages and means were used to describe the participants’ characteristics and their answers for each item of the questionnaire. To assess the normality of quantitative variables, the Kolmogorov–Smirnov test was conducted and the results indicated that *p* value less than 0.05 that means the data were not normally distributed. Consequently, nonparametric statistics were used in this study, namely the scores of knowledge and the variables were compared by using Mann–Whitney test for the items of gender, governance, received training in CPR, and received information about CPR, or Kruskal- Wallis test for the items of age, level of education, and experience. The significance level for all tests was set at *p* ≤ 0.05.

## Results

Out of 220 distributed questionnaires, 200 were returned with a response rate of 90.9%. The Socio-demographic and work characteristics of nurses are presented in Table 1. More than half of the participants were male (56.5%). The mean age of the participants was 29.89 ± 8.58 years, and more than half of the participants had a diploma (58.5%). Just fewer than half (43.5%) had nursing experience of less than five years. A large proportion (59%) of participants had not received training on CPR while more than a half (54.5%) had received information regarding CPR, and an education institution was the major source for this information.

**Table 1:** Socio-demographic and work characteristics of nurses

Item	Characteristics	n (%)
Sex	Male	113 (56.5%)
	Female	87 (43.5%)
Age	Mean ± SD	29.89 ± 8.58
Governance	Hodeidah	100 (50%)
	Taiz	100 (50%)
Level of education	Diploma	117 (58.5%)
	Bachelor	78 (39%)
	Master	5 (2.5%)
Experience	< 5 years	87 (43.5%)
	5–10 years	69 (34.5%)
	> 11 years	44(22%)
Training CPR	Yes	82 (41%)
	No	118(59%)
Received information	Yes	109 (54.5%)
	No	91 (45.5%)
Source of information	Education institution	47 (43.1%)
	Practice	28 (25.7%)
	Self-learning	17 (15.6%)
	Education institution and practice	17 (15.6%)

Table 2 shows that the sum total percentage of correct answers for the questionnaire was (53.65%). Nurses had higher percent correct scores (80.5%), (79.5%),(67%) for the questions “What to do for victim if unresponsive and not breathing normally”, “The ratio of chest compressions to rescue breaths for children”, and “choking during food eating and he can’t cough”, respectively.

**Table 2:** Nurses’ correct and incorrect answers for items of BLS knowledge questionnaire

No	Question	Correct Scores (%)	Incorrect Scores (%)
1	BLS meaning	134 (67%)	66 (33%)
2	What to do for victim if unresponsive and not breathing normally	161 (80.5%)	39 (19.5%)
3	Correct position of hands during chest compressions	101 (50.5%)	99 (49.5%)
4	The depth of chest compressions for adults	103 (51.5%)	97 (48.5%)
5	The ratio of chest compressions to rescue breaths for adults	108 (54%)	92 (46%)
6	The ratio of chest compressions to rescue breaths for children	61 (30.5%)	139 (69.5%)
7	Timing of rescue breaths between the chest compressions	107 (53.5%)	93 (46.5%)
8	The rate of chest compressions	50 (25%)	150 (75%)
9	Position during chest compressions	159 (79.5%)	41 (20.5%)
10	What to do for a victim that is choking while eating and cannot cough	89 (44.5%)	111 (55.5%)
	Total score	1073(53.65%)	927(46.35%)

Table 3 shows the relationship between the demographic characteristics and knowledge scores. There was no relationship between the nurses’ knowledge and the items of sex, age, and years of experience. In contrast, there was a significant relationship between the nurses’ knowledge and the items of governance, level of education, received training in CPR, and received information about CPR.

**Table 3:** Relationship between demographic characteristics and knowledge scores (*n* = 200)

Factors	Group	N	Mean Rank	Median, (IQR)	*P*-value
Gender^a^	Male	113	101.19	5 (4–6)	0.845
	Female	87	99.60	1 (1–2)	
Governance^a^	Hodeida	100	111.70	5 (4–6)	0.005
	Taiz	100	89.30	4 (3–5)	
Received training in CPR^b^	Yes	82	131.59	5 (4–6)	0.000
	No	118	78.89	4 (3–5)	
Received information about CPR^b^	Yes	109	131.09	5 (4–6)	0.000
	No	91	63.86	3 (2–4)	
Age category (years)^b^	< 30	41	110.72	6 (5–6.5)	0.101
	30–39	94	95.55	5 (4–6)	
	≥ 40	65	101.21	5 (4–6)	
Level of education^b^	Diploma	117	87.63	5 (4–6)	0.000
	Bachelor	78	120.51	6 (4–7)	
	Master	5	89.60	5 (3.5–6)	
Experience (years)^b^	< 5	87	105.29	5 (4–6)	0.562
	5–10	69	100.93	5 (4–6)	
	> 10	44	90.35	5 (4–6)	

^a^Mann–Whitney test

^b^Kruskal–Wallis test was conducted at α = 0.05

## Discussion

To our knowledge, this is the first survey of BLS knowledge among nurses in Yemen. In general, the results of this study are consistent with other similar studies performed in various countries with similar socioeconomic hardships to those of Yemen, namely that nurses in underdeveloped countries lack adequate knowledge of BLS procedures. In comparison to the nurses in our study who answered 53.65% of questions correctly, a study among cardiologists in Istanbul found the median percentage of correct answers to be 53% [11]. Medical students at a teaching hospital in Oman had a similar mean score, answering 5.5 questions out of 10 correctly as a whole [7]. In the present study and those in Oman and Istanbul, all participants had a medical background, which may account for the similar results. In contrast, only 33% of general university students in Lebanon said they felt confident in performing CPR [12], the mean score among student-teachers at a University in South Africa was 4.0 out of 12 points [13], and among school teachers in Saudi Arabia there was a sum total of 1387 correct answers given and 1703 incorrect answers given to a knowledge questionnaire [12–14]. These studies from Lebanon, South Africa, and Saudi Arabia were conducted among the general or non-medical population, which may account for the difference in average scores when compared to studies on medical personnel. Finally, a survey of Nurses in Greece found that 25.9% of participants answered 0 questions correctly out of 8 total questions on BLS knowledge, while only 15.5% answered 5 or more questions correctly [15]. This result from nurses is in stark comparison to the present study. One explanation is how recently the persons taking the survey took a training or refresher course on BLS, as only 1.3% of nurses in Greece had taken a BLS course in the preceding 6 months. Taken together, all of these studies point to the fact that medical personnel, who as a whole should have excellent knowledge of BLS and CPR, do not have sufficient knowledge to ensure maximum chances of survival in the case of cardiac arrest or foreign body obstruction of the airway.

The present study demonstrates that there are several important factors associated with higher knowledge scores, namely level of education, having previously received training in CPR, and having previously received information about CPR. This indicates that direct exposure to CPR training and information as well as higher levels of overall medical training are important to obtaining and retaining knowledge of BLS. These results are consistent with studies among cardiologists in Istanbul, medical students in Oman, and teachers in South Africa [7, 11, 13]. However, a study of female teachers in Saudi Arabia found no association between BLS training and knowledge scores, which the study attributed to the fact that many had not had BLS training in the preceding 2 years [14]. One interesting finding of the present study was that Governance was associated with knowledge score. A possible reason for this is that participants from Al-rahida and Al-dimna in the Governance of Taiz are in a rural area without nearby university hospital or BLS training center. In comparison Hodeidah is a more urban environment with a university hospital and training center. This distinction supports the idea of increased BLS knowledge with increased access to training and academic centers and suggests that implementing additional training centers or programs in rural areas would be beneficial.

It was surprising to find that age and years of experience are not associated with knowledge scores. One might assume that with age and years of experience come increased exposure to BLS and improved knowledge, however that does not appear to be the case. This further emphasizes the need for direct BLS training. These results are in comparison to those of non-medical personnel in Ethiopia, which did find significant association between knowledge score and both age and sex [16]. This contrast highlights the differences that can arise among different cultural backgrounds as well as the difference between medical and non-medical persons. Those in the medical field would be more likely to have training and exposure to BLS, and so these factors would play a dominant role in knowledge scores. In non-medical personnel, where training may be less frequent, other factors may play a dominant role in knowledge scores, such as age or sex, depending on the societal norms and influences.

In looking at the questions that nurses most frequently answered correctly or incorrectly, we can see more clearly that there is an important knowledge gap in BLS among nurses. Nurses were more likely to answer questions about basic BLS knowledge correctly, such as “What to do for victim if unresponsive and not breathing normally”. However, for questions that are far more applicable, such as those dealing with the specifics of how to perform CPR or what to do for a victim that is choking and unable to breath, the correct response rates fell far below the average. This finding indicates that specific knowledge of key BLS procedures resulting in “high-quality” CPR is lacking. Thus, while the overall correct response rate for nurses in Yemen may be similar to those from other countries, the most important aspects for saving a life in the case of a cardiac arrest may be absent when it is performed by the nurses included in the study [17]. This emphasizes the need for improved BLS knowledge among nurses in Yemen. It can be difficult to grasp the consequences of the conflict in Yemen. A survey of Medical Schools in Iraq found deans frequently believed violent conflict had negatively impacted student performance [18]. As a result of conflict medical students themselves felt their training had been impaired, frequently experienced threats to their lives or health, wanted to drop out 26% of the time, and were uncertain about dropping out another 25% of the time. These findings highlight some of the hardships that conflict can impart on medical training and the healthcare system.

Recent studies estimate that out-of-hospital cardiac arrest accounts for up to 10% of total mortality in developing countries [19]. Ensuring adequate BLS knowledge in medical personnel is a first step in the chain of survival for preventing cardiac death. In a country like Yemen, where active warfare has resulted in an estimated 377,000 deaths by the end of 2021, 154,000 of which were a result of direct combat and violence, adequate BLS knowledge becomes even more pivotal in order to prevent death [20]. The present study shows that there is a critical knowledge gap in Yemen, and that significant improvements can be made in the knowledge of BLS among nurses, specifically when it comes to the applicable aspects of BLS. It also shows that focusing on increased access to BLS information and increased emphasis on direct BLS training can yield significant positive results. As a result of improved BLS knowledge, many lives could be saved, both those that are at risk because of the conflict in Yemen as well as those who have more natural maladies. The subject of implementing BLS training has received worldwide attention and there is plenty of data already published to guide an implementation strategy [21, 22]. Providing recommendations for improving BLS knowledge in Yemen in a cost effective and efficient manner could be a good topic for future research. On the contrary, the fact that BLS knowledge among nurses in Yemen is comparable to that of other developing countries on average points to the resilience of the Yemen medical community to the unique socioeconomic hardships in Yemen including war, casualties, famine, an exodus of medical personnel, internal displacement of peoples, and socioeconomic collapse [23]. These hardships lead to increases in trauma-related mortality, infectious diseases, malnutrition, and non-communicable disease, along with worsening access to basic health services such as neonatal maternal healthcare which impacts neonatal and maternal mortality rates [24]. When combined with less trained medical personnel due to attacks on healthcare facilities or emigration such as in Yemen, the end result is increasing mortality and morbidity with decreasing quality of healthcare services [25]. All of these factors contribute to an increased demand for adequate BLS while also diminishing the availability of those with adequate BLS knowledge. This study highlights this knowledge gap, and hopefully will lead to an appropriate response among the medical community in Yemen and around the world to help save as many lives as possible.

### Limitations of the study

There are several limitations of this study. As a cross sectional survey conducted among nurses in two governances of Yemen, this study may not be generalizable to other populations and is subject to selection bias and non-response bias. Similarly, the unique socioeconomic factors in Yemen, especially open conflict and active warfare, present a unique challenge to nurses and the society at large. These factors may impact the concordance of results with those from other countries or from other parts of Yemen. This study only assessed the theoretical knowledge of participants and did not assess the practical skills of participants in performing basic life support.

### Relevance to clinical practice

The results of the present study highlight the importance of continuous evaluation of nurses’ knowledge of BLS which will increase their awareness of updated guidelines. In addition, due to the inadequate nurses’ knowledge found in this study, we recommend the organization and implementation of regular in-service education and training programs for nurses and other health care providers regarding BLS, especially in areas near to conflict in Yemen.

## Conclusion

The findings of this study show that BLS knowledge among nurses in Yemen is comparable to other underdeveloped countries on average, but that the knowledge of critical aspects of BLS necessary to save a life are inadequate given the increased demand for BLS in the face of active warfare. Yemen faces unique socioeconomic hardships due to violent conflict in the country, and in light of this, the findings of this study are encouraging but also highlight room for improvement and the possibility of saving many lives. Efficient solutions to this problem such as providing pamphlets with BLS information on them, online BLS training, or regular in person BLS training seminars could have a huge impact on BLS knowledge among nurses in Yemen. Future research and efforts should be aimed at implementing strategies to improve BLS knowledge among nurses in Yemen, with a long-term goal of improving BLS knowledge among the non-medical population as well to decrease the burden associated with sudden cardiac death as well as cardiac death from the trauma of warfare.

## Data Availability

The data that were generated and analyzed in this study are mostly included within the published article. However, source material and the raw datasets are available from the corresponding author upon request.

## Abbreviations

BLS: Basic life support
CPR: Cardiopulmonary resuscitation
SPSS: Statistical Packages for Social Sciences
